# Postnatal Allergic Inhalation Induces Glial Inflammation in the Olfactory Bulb and Leads to Autism-Like Traits in Mice

**DOI:** 10.3390/ijms251910464

**Published:** 2024-09-28

**Authors:** Eizo Tanaka, Ryo Yamasaki, Ban-yu Saitoh, Amina Abdelhadi, Satoshi Nagata, Sato Yoshidomi, Yuka Inoue, Koichiro Matsumoto, Jun-ichi Kira, Noriko Isobe

**Affiliations:** 1 Department of Neurology, Neurological Institute, Graduate School of Medical Sciences, Kyushu University, 3-1-1 Maidashi, Higashi-ku, Fukuoka 812-8582, Japan; 2 Department of Neurology, Miyazaki Prefectural Miyazaki Hospital, 5-30 Kita-Takamatsu-Cho, Miyazaki 880-8510, Japan; 3 Department of Neurology, Himeno Hospital, 2316 Oaza-Nishiro, Hirokawa-machi, Yame-gun, Fukuoka 834-0115, Japan; 4 Department of Medical Microbiology and Immunology, Faculty of Medicine, Zagazig University, Zagazig 44519, Al-Sharqia Governorate, Egypt; 5 Clinical Education Center, Kyushu University Hospital, 3-1-1 Maidashi, Higashi-ku, Fukuoka 812-8582, Japan; 6 Department of Anesthesiology and Critical Care Medicine, Graduate School of Medical Sciences, Kyushu University, 3-1-1 Maidashi, Higashi-ku, Fukuoka 812-8582, Japan; 7 Division of Respirology, Department of Medicine, Fukuoka Dental College, 2-15-1 Tamura, Sawara-ku, Fukuoka 814-0193, Japan; 8 Translational Neuroscience Center, Graduate School of Medicine, and School of Pharmacy at Fukuoka, International University of Health and Welfare, 137-1 Enokizu, Okawa 831-8501, Japan; 9 Department of Neurology, Brain and Nerve Center, Fukuoka Central Hospital, 2-6-11 Yakuin, Chuo-ku, Fukuoka 810-0022, Japan

**Keywords:** allergic rhinitis, autism spectrum disorder, olfactory bulb, eosinophil, glial inflammation, medial prefrontal cortex

## Abstract

Autism spectrum disorder (ASD) is one of the most prevalent neurodevelopmental disorders. To explore its pathophysiology, we investigated the association between neonatal allergic exposure and behavioral changes. Adult female C57BL/6J mice were immunized with adjuvant (aluminum hydroxide) or ovalbumin emulsified with adjuvant. After immunization, the mice were mated, and offspring were born at full term. The postnatal dams and infants were then simultaneously exposed to an allergen (ovalbumin) or vehicle via inhalation. After weaning, behavioral testing and histopathological analyses were conducted on male offspring. Compared with the vehicle-exposed offspring, the ovalbumin-exposed offspring had decreased sociability and increased repetitive behavior, thus representing an ASD-like phenotype in mice. Moreover, histopathological analyses revealed that the ovalbumin-exposed mice had increased astroglial, microglial, and eosinophilic infiltration in the olfactory bulb, as well as increased eosinophils in the nasal mucosa. The ovalbumin-exposed mice also had decreased dendritic spine density and a lower proportion of mature spines, suggesting the impairment of stimulus-induced synaptogenesis. In conclusion, postnatal allergic exposure induced an ASD-like phenotype, as well as allergic rhinitis, which was followed by glial inflammation in the olfactory bulb parenchyma.

## 1. Introduction

Autism spectrum disorder (ASD) is one of the most prevalent neurodevelopmental disorders [1,2]. The prevalence estimates of ASD have increased worldwide over time, and estimated prevalences differ across distinct geographic regions and ethnicities [3]. A recent report indicated that by 8 years of age, the frequency of ASD in the United States is 1 in 54 children, and ASD predominantly affects boys more than girls at a ratio of approximately 4:1 [4]. Multiple factors contribute to ASD pathogenesis: genetic susceptibility, internal factors such as autoimmunity (including allergic conditions), and environmental factors such as infections, toxic substances, environmental pollution, and malnutrition [1,5].

The increase in individuals with ASD coincides with a worldwide rise in the prevalence of allergic disorders, of which allergic rhinitis is the most common [6,7]. It is estimated that allergic rhinitis affects approximately 24.8% of US children and 28.2% of Japanese children [8,9]. Furthermore, numerous cross-sectional and longitudinal investigations have demonstrated an epidemiological relationship between allergies and ASD [10,11,12,13]. Neuroinflammation, which can impair memory, mood, and cognitive functions, can be caused by a variety of immune modulators and cytokines that are induced by allergic rhinitis [14]. Nonetheless, pathways that may link nasal inflammation with neuropsychiatric diseases remain unclear.

It is crucial to comprehend how nasal allergic irritation impacts the structure and functioning of the brain. The association between allergic exposure and the nervous system is a major research focus for our group. In a previous study, we found that allergic exposure in mice induced microglial and astroglial activation in the spine, leading to neuropathic pain [15]. We also demonstrated that in the short term (30 days), early postnatal allergic airway inflammation does not induce ASD-like behaviors. However, allergic exposure-induced microglial changes and an ASD-like phenotype appear by postnatal day (P) 70, when mice reach their adolescent period [16]. By contrast, the human ASD phenotype usually occurs early in life—before the age of 3 years—during which time a large amount of synaptogenesis and microglial synaptic pruning occurs [17,18]. This discordance between the natural course of ASD and the findings in our previous study may be caused by our study procedures, at least in part because the mice were isolated from their dams during stressful intranasal allergic exposure, and the frequency of allergic exposure (twice weekly) may have been insufficient to induce allergic changes in the central nervous system (CNS). Therefore, in the present study, we hypothesized that a less stressful but more frequent allergy induction that more closely resembles environmental exposure, rather than long-term intranasal allergic exposure, may be required to avoid any residual effects on the behavior of the offspring. To explore the association between allergic exposure and ASD-like behaviors, we prenatally immunized dam mice with adjuvant (aluminum hydroxide [alum]) only or ovalbumin (OVA) emulsified with adjuvant, and we postnatally exposed both dam and offspring mice (simultaneously) to aerosolized 2.5% OVA. We found that postnatal allergic exposure induced ASD-like behavioral changes, as well as allergic rhinitis, with glial inflammation in the olfactory bulb (OB) parenchyma.

## 2. Results

### 2.1. Gradual Inducement of Airway Allergy in Dams

First, we investigated the lung histopathology of the dams in each group to observe whether there were asthmatic changes as a result of allergic airway inflammation. Compared with the non-allergic group, we noted bronchial inflammatory cell infiltration, increased PAS-positive mucus production, and increased eosinophils in the broncho-alveolar lavage fluid (BALF) in the mildly allergic group and more severely in the allergic group. These findings are consistent with allergic asthma, and as the intensity of allergy induction increased, there were increased allergic inflammation findings (Supplementary Figure S1).

### 2.2. Mild Allergy-Induced ASD-like Behavioral Changes in the Offspring

**Figure 1:**
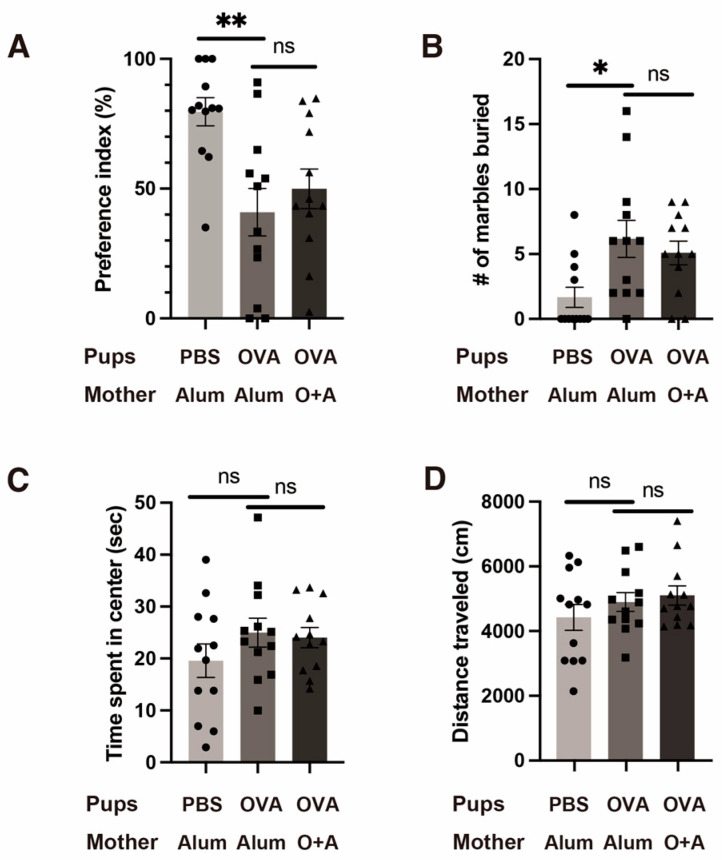
Behavioral tests for detecting ASD-like traits in each group of mice. (**A**) The three-chamber test for assessing sociability deficits. Sociability was measured using the preference index, which represents a preference for the chamber with a novel mouse versus the empty chamber. (**B**) The marble burying test for assessing repetitive/obsessive behavior. (**C**,**D**) The open field test for assessing anxiety (**C**) and locomotion ability (**D**). Less time spent in the center represents higher anxiety. *n* = 12 per group. All values are shown as the mean ± SEM. ns = not significant, * *p* < 0.05, ** *p* < 0.01 (Tukey’s multiple comparison test).

Next, we performed behavioral testing on the pups. In the three-chamber test, the preference index (which reflects sociability in mice) was lower in the mildly allergic group than in the non-allergic group (*p* < 0.01; Figure 1A). Notably, however, there were no significant differences in the preference index between the mildly allergic and allergic groups (*p* = 0.68). These results suggest that simple allergic exposure in the dam and pups, rather than intensive maternal allergic exposure with antigen sensitization, is sufficient to induce impairments in pup sociability.

Similarly, in the marble burying test, which is used to assess repetitive and obsessive behavior, the mildly allergic group buried significantly more marbles than the non-allergic group (*p* < 0.05). There were no significant differences between the mildly allergic and allergic groups (*p* = 0.76; Figure 1B).

In the open field test, which is used to assess anxiety behaviors, there was a tendency toward more time spent in the center, reflecting less anxiety, in the mildly allergic group than in the non-allergic group; however, this difference did not reach significance among the three groups (*p* = 0.35; Figure 1C). Similarly, there were no significant differences in the distance traveled in this test (*p* = 0.57; Figure 1D).

In summary, OVA aerosol inhalation-induced allergic exposure caused sociability impairments and repetitive behaviors, both of which are core symptoms of ASD. Maternal pre-sensitization did not affect the behavioral results of the pups. Given that pup behavioral changes were caused by the inhalation of OVA by pups only, we conducted all further experiments using just two groups: the non-allergic group (hereafter referred to as the PBS group) and the mildly allergic group (hereafter referred to as the OVA group).

### 2.3. Effects of Maternal Nursing Behavior

To evaluate whether maternal nursing behavior affected adolescent pup behavior, we compared maternal nursing behaviors between the PBS and OVA groups. There were no differences in the four nursing behaviors (licking/grooming, nursing, off pups, and self-grooming) between the dams of the two groups. These results suggest that any behavioral changes in the pups were not caused by maternal nursing behavior (Supplementary Figure S2).

### 2.4. Allergic Rhinitis in OVA Group According to Histopathological Examination of Lung and Nasal Mucosa

**Figure 2:**
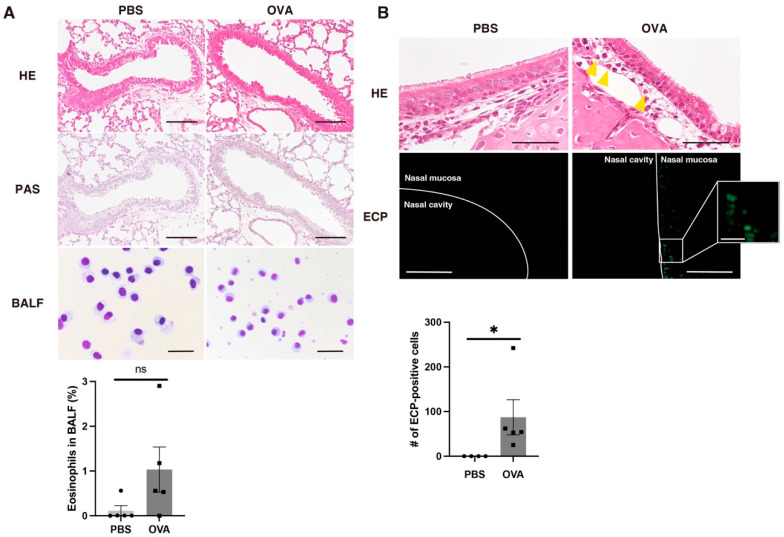
(**A**) Lung histopathological analyses of offspring exposed to OVA or PBS. In HE and PAS staining, there were no obvious increases in granulocyte infiltration or mucus production (i.e., asthmatic changes) in the OVA and PBS groups. BALF sediment analyses showed no significant increases in eosinophils in either group. Scale bars: HE and PAS, 100 µm; BALF, 30 µm. (**B**) Histopathological analyses of the nasal mucosa. In HE staining, some granulocyte infiltration into the nasal mucosa (yellow arrowheads) was observed in the OVA group but not in the PBS group. These infiltrated granulocytes were positive for ECP, indicating that allergen inhalation induces tissue eosinophilia. Scale bars: outside the inset, 50 µm; inside the inset, 10 µm. *n* = 4 for the PBS group in (**B**), and *n* = 5 per group for all others. All values are shown as the mean ± SEM. ns = not significant, * *p* < 0.05, (Mann–Whitney *U* test).

We then investigated the histopathological changes in the pups. There was no bronchial inflammatory infiltration or mucus production in the lungs (i.e., there were no asthmatic findings) in the PBS or OVA groups (Figure 2A). In the BALF samples, almost all cells were alveolar macrophages, although a few eosinophils were also observed in the OVA group. These findings suggest that there were almost no differences in the lung allergic changes between the two groups. This may be because neither group was immunized using adjuvant-emulsified antigens. Next, we focused on another airway allergy: allergic rhinitis. Allergic rhinitis often precedes the occurrence of allergic asthma, and it has a higher worldwide prevalence than allergic asthma [19,20]. Mild granulocyte infiltration was observed in the nasal mucosa of the OVA group compared with the PBS group (Figure 2B). We then conducted immunohistochemistry for eosinophil cationic protein (ECP) in the nasal mucosa to differentiate infiltrated eosinophils from other granulocytes. We identified a few ECP-positive eosinophils that had infiltrated into the nasal mucosa of OVA mice (Figure 2B). In summary, mild allergic rhinitis but no allergic asthma occurred in mice exposed to allergens without antigen sensitization.

### 2.5. Allergic Rhinitis-Induced Glial Inflammation in the OB

**Figure 3:**
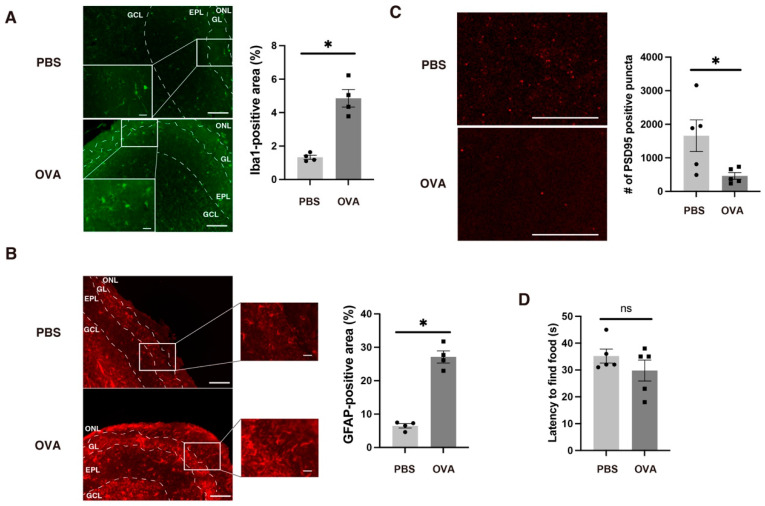
Allergic rhinitis induced glial inflammation and synaptic loss in the OBs of offspring. (**A**) Ionized calcium-binding adapter molecule 1 (Iba1)-positive microglia were increased on the OB surface (ONL + GL + EPL) of OVA-exposed offspring. *n* = 4 per group. Scale bars: outside the inset, 100 µm; inside the inset, 20 µm. (**B**) GFAP-positive astrocytes were also increased on the OB surface of OVA-exposed offspring. *n* = 4 per group. Scale bars: outside the inset, 100 µm; inside the inset, 20 µm. (**C**) PSD95-positive postsynaptic punctum numbers per microscopic view were decreased on the OB surface of OVA-exposed offspring. *n* = 5 per group. Scale bars: 20 µm. (**D**) The buried food pellet test for detecting olfactory impairment. Although there was synaptic loss in OVA-exposed offspring, no robust olfactory impairments were detected in these mice. *n* = 5 per group. All values are shown as the mean ± SEM. ns = not significant, * *p* < 0.05, (Mann–Whitney *U* test).

A previous study demonstrated that lipopolysaccharide-induced rhinitis results in microglial and astroglial activation and synaptic loss in the surface layers of the OB [21]. We therefore examined the microglia and astroglia located on the surface (i.e., olfactory nerve layer [ONL], glomerular layer [GL], and external plexiform layer [EPL]) of the OB. Using immunohistochemistry, the ionized calcium-binding adapter molecule 1 (Iba1)- and glial fibrillary acidic protein (GFAP)-positive areas on the OB surface were increased in the OVA group compared with those in the PBS group (*p* < 0.05 for Iba1, Figure 3A; *p* < 0.05 for GFAP, Figure 3B). We also performed immunohistochemistry for PSD95, a postsynaptic protein, in the external plexiform layer; there were fewer PSD95-positive puncta in the OVA group than in the PBS group (*p* < 0.01; Figure 3C). We then performed additional behavioral testing—the buried food test—to identify whether any olfaction loss was caused by OB synaptic loss. However, there was no difference in the latency to find food between the two groups (*p* = 0.67; Figure 3D), indicating no major olfaction loss in the OVA group.

### 2.6. Eosinophilic Protein Upregulation in OVA Group in Microarray Analysis of OB

To elucidate any ASD phenotype-inducing gene expression changes in the OB, we conducted a microarray analysis of bulk OB tissue to identify differentially expressed genes, defined as a *z*-score ≥ 2 or ≤ −2. The DAVID was used to identify affected Gene Ontology (GO) biological processes. Of the many olfaction- or chemical sensation-related processes that were affected by OVA inhalation, there was an upregulation of mucosal innate immune response genes. These upregulated genes included alpha-defensin, which was reported to be upregulated in activated eosinophils [22], and both *Ear4* and *Rnase2b*, which are eosinophilic granular proteins. Although not included in the aforementioned biological process gene set, *Ccr3*, which is mainly used as an eosinophilic cellular marker, was another upregulated gene.

**Figure 4:**
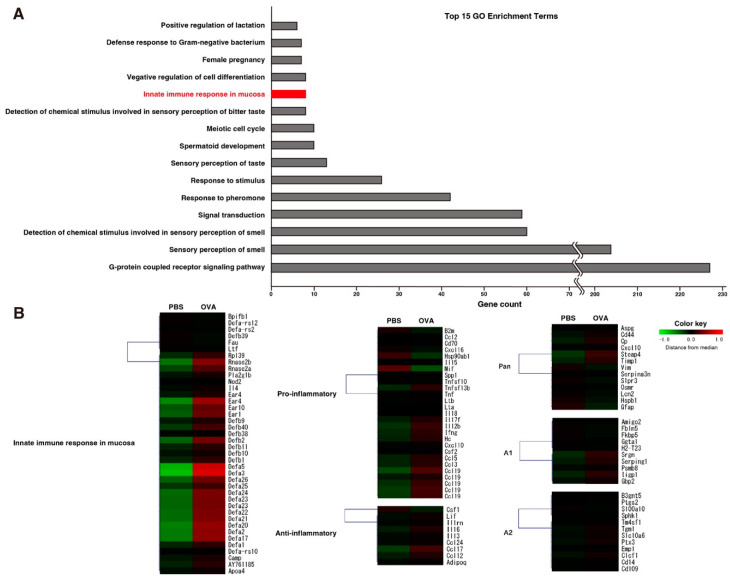
A GO analysis and heatmap/cluster analysis of differentially expressed genes detected by whole OB bulk microarray analysis. (**A**) The top 15 GO enrichment terms, ranked by the gene counts of differentially expressed genes. Although most of the terms were related to olfactory functions, the innate immune response in mucosa terms included differentially expressed genes that were related to activated eosinophils. (**B**) A heatmap/cluster analysis of the following GO terms: innate immune response in mucosa; proinflammatory and anti-inflammatory genes; and astrocyte-related pan-reactive, A1—specific, and A2—specific genes. Although there were no alterations in astrocyte-related genes or inflammatory genes, genes related to innate immune response in mucosa had increased expression in the OB of OVA—exposed mice; most of these genes were related to activated eosinophils.

We subsequently conducted a heatmap/cluster analysis of mucosal innate immune response genes, including both pro- and anti-inflammatory genes [23] and astroglia-related genes [24]. Although no obvious changes were noted in glial- or inflammation-related genes, upregulated expression was detected in mucosal innate immune response genes, similar to the findings of our GO analyses. Figure 4 shows the top 15 GO enrichment processes (Figure 4A) and heatmap results (Figure 4B). Taken together, these results suggest that OB glial inflammation is caused by the eosinophilic infiltration of the OB.

**Figure 5:**
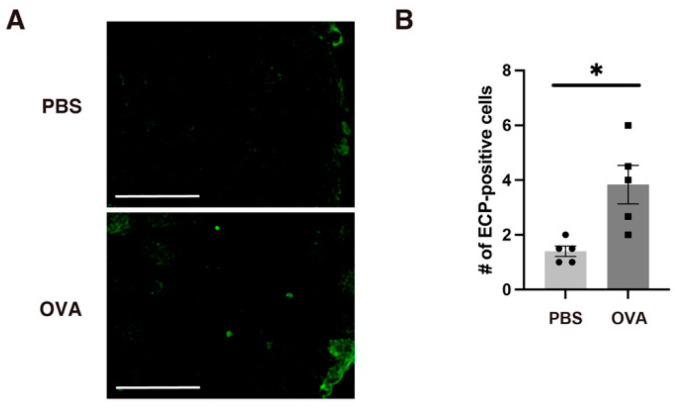
Allergic rhinitis induced OB tissue eosinophilia in OVA-exposed offspring. ECP-positive cells on the surface of the OB were increased. Representative microscopic views (**A**) and quantified numbers (**B**) are shown. *n* = 5 per group. Scale bars: 50 µm. All values are shown as the mean ± SEM. * *p* < 0.05 (Mann–Whitney *U* test).

To investigate whether allergic rhinitis induced eosinophilic inflammation in the OB, we stained the OB for ECP. Similar to Iba1 and GFAP, ECP immunoreactivity was observed in the surface layers of the OB in the OVA group (Figure 5A,B). These results indicate that allergic rhinitis may result in eosinophilic infiltration (and some proinflammatory factor diffusion) to the OB, which is located near the nasal mucosa, and may then proceed to OB glial inflammation (including microglia and astrocytes).

### 2.7. OB Glial Inflammation-Induced Synaptic Immaturity in the Medial Prefrontal Cortex

**Figure 6:**
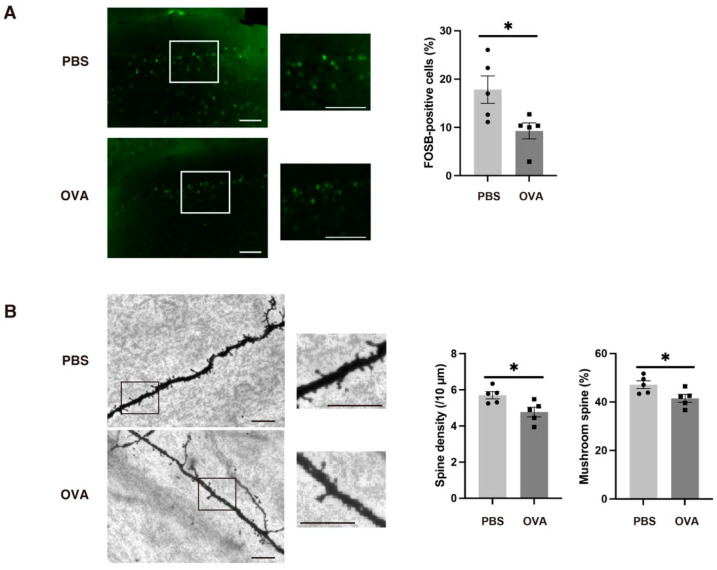
Allergic rhinitis led to decreased neural activity transmission to the medial prefrontal cortex and impaired synaptic maturation, which may be the origin of the observed ASD-like traits. (**A**) FOSB-positive neurons were decreased in the medial prefrontal cortex of OVA-exposed offspring. *n* = 5 per group. Scale bars: 100 µm. (**B**) In Golgi–Cox staining, both the spine density and the percentage of mature mushroom spines were decreased in OVA-exposed offspring. *n* = 5 per group. Scale bars: 10 µm. Each image on the right shows the magnified area of the rectangle in the left image. All values are shown as the mean ± SEM. * *p* < 0.05 (Mann–Whitney *U* test).

We then focused on the medial prefrontal cortex (mPFC) because allergic rhinitis has been reported to impair neural connectivity between the OB and mPFC [25]. We performed immunohistochemistry for FBJ murine osteosarcoma viral oncogene homolog B (FOSB), which is one of the immediate early genes that reflect neural activity. There were fewer FOSB-positive neurons in the mPFC of the OVA group compared with the PBS group (Figure 6A). We further examined the mPFC apical dendrite synaptic density and performed morphological analyses using Golgi staining, because the dendritic spines of these regions were reported to be affected [26]. Apical dendrite synaptic density was lower, and there were fewer mature spines (i.e., mushroom forms) in the OVA group than in the PBS group (Figure 6B). By contrast, there was no difference between the two groups in the Iba1-positive area of the mPFC.

**Figure 7:**
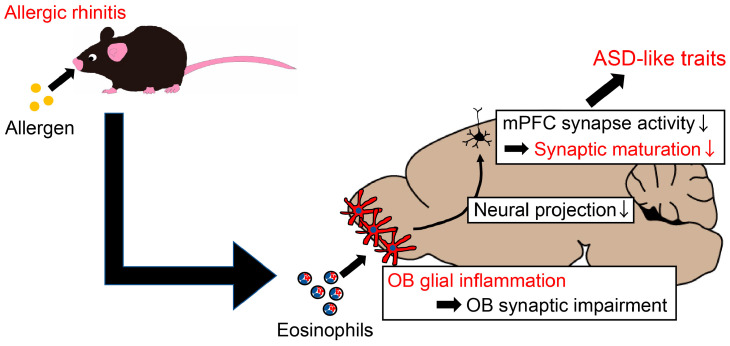
Schematic illustration of mechanisms by which mild allergic airway exposure may cause ASD-like behavioral changes in offspring mice.

In summary, our findings indicate that allergic rhinitis induces eosinophilic infiltration in the surface layers of the OB. The resulting secondary glial inflammation and synaptic loss may then lead to inappropriate neural projections to the mPFC, resulting in immature synapses and an ASD-like phenotype (Figure 7).

## 3. Discussion

Both ASD and allergic diseases occur early in life, have negative impacts on the quality of life, impose significant economic burdens, and have increasing global prevalences [3,6,7,27]. Although the exact causes of ASD are still not fully understood, a complex interplay among genetic, epigenetic, and environmental factors may play a role. Maternal immune activation by the infectious and autoimmune status is one environmental factor associated with ASD occurrence [28]. An association between maternal allergic status during pregnancy and ASD in offspring has also been observed in a clinical study [29]. Using animal models, our research group and many others are actively exploring the processes by which maternal immune activation and maternal allergy may affect offspring brain development and result in neurodevelopmental disorders [16,30,31,32,33]. In the present study, we therefore induced maternal allergy by OVA sensitization, followed by postnatal OVA aerosol inhalation by the dam and their offspring (simultaneously).

In accordance with the results of Kim et al. [34], our histopathological examination of maternal lungs confirmed very mild asthmatic changes in the mildly allergic group, which did not receive allergen sensitization, and more severe typical changes in the allergic group; these findings indicate gradual maternal allergic induction. Moreover, mild allergic rhinitis was noted in the offspring of the mildly allergic group compared with the non-allergic group. In addition, the mildly allergic group displayed impaired sociability and repetitive behaviors, and there were no significant behavioral differences between the mildly allergic and allergic groups. These results imply that intensive maternal allergic exposure with antigen sensitization is not required for ASD occurrence, and allergic exposure in pups is sufficient to induce sociability impairments. These novel findings are slightly different from those in our previous study, which concluded that prolonged and strong allergic exposure (70 days) and previous sensitization are required for the induction of ASD-like behavior in offspring [16]. The inconsistency between the present study and our previous study may be attributed to the different protocols used for inducing allergy in the animals.

Our finding of the allergic rhinitis-induced eosinophilic infiltration of the OB, which is part of the CNS, has never previously been reported. The CNS is an immune-privileged area, and it is difficult for peripheral inflammation to spread to this region [35,36]. There are two possible pathways by which eosinophils may infiltrate the OB: (1) circulating eosinophils in the peripheral blood are attracted to the OB by a humoral homing factor as a result of glial inflammation in the OB, or (2) eosinophils infiltrate the OB via a simple diffusion mechanism from the olfactory mucosa through the cribriform plate—a hole in the skull bone—and the olfactory nerve. The latter explanation seems the most likely, because glial inflammation and eosinophilic infiltration are the strongest in the OB—the closest brain region to the nasal mucosa—and weaker in other regions. Indeed, it has been suggested that certain viruses and toxic substances may invade the CNS through the olfactory mucosa and the OB [37]. This infiltration pathway may thus work as an immune gateway to the CNS. However, further studies are needed to confirm this hypothesis.

In the present study, we detected inflammatory responses in the OB as increased immunoreactivity against microglial Iba1 and astroglial GFAP. Microglia are the major glial cells in the CNS and are activated during insults to the CNS. These activated cells subsequently clear tissue debris, produce inflammatory cytokines, and phagocytose synapses with the aid of complement factors [38]. Similarly, activated reactive astrocytes have been implicated in phagocytosis [39], synaptic reorganization [40], and proinflammatory mediator release [41] during CNS regeneration. Both microgliosis and astrogliosis may thus potentially cause long-term CNS injury by interfering with axon regeneration and dendritic reconnection. The absence of receptor axons and synapses in the OB stimulates microglial cells and encourages the invasion of monocytes to clear away localized debris. It has been reported that LPS-induced rhinitis activates microglia and astrocytes in the OB lateral portion of the external plexiform layer [21]. Consistent with this finding, we revealed a decrease in PSD95-positive puncta (i.e., synapses) in the OB of the OVA group. Kim et al. [42] also reported that microgliosis and astrogliosis are induced in the mouse OB after rhinitis caused by intranasal exposure to Triton X-100. Taken together, these findings imply that, in response to a nasal insult, the synapses of second-order neurons and interneurons of the OB are negatively affected by the loss or degeneration of olfactory neurons and/or subsequent glial activation in the OB.

Severe allergic rhinitis frequently causes olfactory impairment through olfactory nerve loss [43,44]. Although ASD patients reportedly have impaired olfactory functions [45], we did not detect any changes in olfaction in our allergic rhinitis mice with ASD-like behaviors. There are several possible reasons for this lack of detected olfactory impairment in our model. For example, it may be that the induced allergic rhinitis—and associated damage to the nasal mucosa and OB—was too mild to induce olfactory impairment. Alternatively, the buried food test, which is a commonly performed olfactory test, may detect gross olfactory impairment but not the fine olfactory discrimination needed for social interaction.

In the present study, there were fewer FOSB-positive cells in the mPFC in the OVA group than in the PBS group. FOSB is an immediate early gene that reflects neural or synaptic activity [46]. Many clinical and preclinical studies have reported on morphological and functional abnormalities in several brain regions, including the PFC, in ASD [26,47]. Moreover, there is robust neural connectivity between the PFC and OB, and this connectivity is reportedly impaired by allergic rhinitis [25]. It has also been reported that c-Fos-positive cells in the mPFC are decreased in a mouse model of maternal immune activation [48].

Dendritic spines are responsible for many neuropsychiatric disorders, especially those associated with cognitive deficits. Research on post-mortem tissue from people with ASD has revealed both decreased and elevated spine densities in cortical neurons under various circumstances [26,49,50]. In the present study, we observed declines in both the total spine density and the mature spine numbers in the mPFC of OVA mice. Furthermore, the reduced FOSB immunoreactivity in the mPFC suggests that the neural inputs reaching the mPFC may be reduced because of synaptic damage in the OB. Given that spine formation and maturation may be facilitated by neural inputs reaching the spines [51,52], it is possible that this decrease in neural inputs leads to the histopathological findings of decreased spine density and maturation in mPFC apical dendrites, which are also seen in ASD pathology. For example, in the human mPFC or anterior cingulate cortex, decreased spine density has been reported [26,53], and similar results were noted in an animal ASD model of environmental factors [54].

Our study has several limitations. First, all dams were treated with intraperitoneal alum to examine the effects of prenatal maternal sensitization on allergic exposure. Alum is a T helper 2-skewing adjuvant [55]; it may thus affect the immune profiles of dams and pups and result in behavioral changes. Therefore, it remains unclear whether the observed behavioral changes would also occur in pups whose dams did not receive any prior treatment. These analyses are beyond the scope of our study and require further examination.

Second, as we previously remarked, although the buried food pellet test is frequently used to test olfaction in mice, it can only detect gross olfactory ability and is not intended for fine olfactory discrimination, which is important for mice sociability. However, the affected external plexiform layer on the OB surface is reportedly important for the fine discrimination of similar odors [56].

Third, although olfaction is relatively important for mice in terms of sociability and perception, it is not as important for humans. Therefore, human samples are needed to explore whether our theory is also applicable to human ASD. Further studies are also needed to clarify the pathomechanisms underlying our study model.

Finally, one of the novel findings in our study is that allergic rhinitis caused eosinophilic infiltration to the OB, which is located near the nasal mucosa. However, the pathway through which eosinophils infiltrate the OB was not fully addressed. We speculate that eosinophils may infiltrate the OB from the nasal mucosa through diffusion-like mechanisms along the olfactory nerve and through the cribriform plate. This hypothesis cannot be confirmed by the present findings, and further investigation is needed.

## 4. Materials and Methods

### 4.1. Ethics

All animal care and experimental procedures were conducted according to institutional guidelines and were approved by the Kyushu University ethics committee (reference numbers: A30-112-2 and A22-188-0).

### 4.2. Animals

Wild-type C57BL/6J male and female mice aged 10–12 weeks were purchased from Japan SLC (Hamamatsu, Japan). The C57BL/6J mice were then bred in our laboratory. All mice were maintained under specific pathogen-free conditions and were provided with food and water ad libitum. The animal room was kept at a constant temperature (20–24 °C) with modest humidity and a 12 h light/dark cycle.

### 4.3. Allergic Sensitization and Exposure

**Figure 8:**
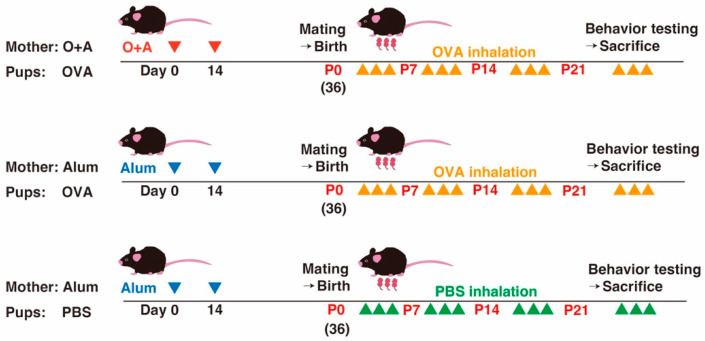
Experimental protocol for simultaneous induction of allergies in dams and their offspring. Red and blue arrowheads indicate intraperitoneal injection of OVA emulsified with alum or of alum only, respectively, for prior allergen sensitization. Yellow and green arrowheads indicate 30 min inhalation of aerosol comprising either 2.5% OVA dissolved in PBS or PBS only, respectively, three times per week. After weaning at P21 to P28, behavioral tests were conducted, and mice were euthanized for histopathological analyses.

A summary of the experimental procedures is shown in Figure 8. The protocol used to induce allergy in the offspring was based on a previously reported method [57] with slight modifications. Specifically, to make the allergic exposure intensity milder, we limited the frequency of exposure, comprising 30 min of inhalation, to three times per week. Adult female mice were intraperitoneally immunized with 50 mg chicken OVA (Grade V, A5503; Sigma-Aldrich, St. Louis, MO, USA), emulsified with 1 mg alum (Alu-Gel-S 12261; Serva, Heidelberg, Germany) or alum only. This immunization was conducted twice at an interval of 2 weeks. After the second immunization, adult female mice were mated with adult male mice, and the offspring were born at full term. Each postnatal dam that was immunized with OVA and alum was simultaneously exposed, together with their offspring (allergic group), to aerosolized 2.5% OVA diluted with phosphate-buffered saline (PBS) for 30 min, three times per week for 3 weeks. Half of the dams immunized with alum only and their offspring were similarly exposed to aerosolized 2.5% OVA (mildly allergic group), whereas the other half were exposed to aerosolized PBS only (non-allergic group). The chamber for allergic exposure comprised a 35 × 30 × 30 cm Plexiglas container that had capacity for two mouse home cages during one exposure challenge. The chamber was connected to an aerosol generator (Single-JET 9302; TSI, Shoreview, MN, USA), which breaks PBS or 2.5% OVA into minute particles that can easily reach the lungs of both the dam and the pup. Based on a previous report [58], the allergic group was considered a classical and definite allergic exposure group. The mildly allergic group was considered a milder allergic exposure group, and because the dams did not receive prior sensitization with the allergen, they were also regarded as the sensitization procedure control. The non-allergic group did not receive any allergen and was thus considered the exposure control. From P21 to P28, the mice were weaned, tested for behavior, and then euthanized for histopathological and biochemical analyses. The offspring received aerosolized exposure until their behavioral testing and euthanasia. All analyses of offspring were conducted on male mice only because, epidemiologically, ASD occurrence has a male predominance.

### 4.4. Three-Chamber Test

A subject mouse was placed in the middle chamber of the testing apparatus, which comprised three empty chambers (20 × 40 cm) divided by clear Plexiglas walls. The mouse was then allowed to move freely within all three chambers over a 10 min habituation period. Next, an empty wire cage was placed in one outside chamber (non-social chamber), and a wire cage containing a stranger mouse was placed in another outside chamber (social chamber). The subject mouse was then allowed to move freely for another 10 min. Automated video tracking software (SMART v.2.3.05; Panlab Harvard Apparatus, Holliston, MA, USA) was used to record the time spent in each chamber. Sociability was quantified and presented as follows [59]: Preference index = (total time in social chamber/[total time in social chamber + non-social chamber]) × 100%.

### 4.5. Marble Burying Test

A subject mouse was habituated in a 31 × 20 × 16 cm cage containing chipped wood bedding (4 cm deep) for 10 min. After habituation, the mouse was returned to its cage, and 20 black marbles were distributed on the bedding of the experimental cage. The mouse was again placed in the experimental cage for 10 min, and the number of marbles that the mouse buried was manually counted.

### 4.6. Open Field Test

A subject mouse was placed in the center of a 50 × 50 cm open field and allowed to move freely for 10 min. The abovementioned automated video tracking software was used to track the distance traveled and to quantify the time spent in the center (defined as a 16.8 × 16.8 cm square in the center of the open field).

### 4.7. Buried Food Pellet Test

The protocol for the buried food pellet test was similar to that previously reported [60]. In brief, the mice were deprived of food pellets overnight from 5:00 p.m. on the day before the test to 8:00 a.m. on the day of the test. The mice had free access to drinking water. On the day of the analysis, one food pellet was buried under the thick bedding of the cage, and the pellet was completely covered. Each test mouse was placed in the cage, and the time taken for the mouse to find and begin to eat the pellet was measured using a stopwatch.

### 4.8. Maternal Behavior

Maternal nursing behavior was assessed as previously reported [61,62]. Briefly, 30 min of behavioral testing was recorded using a video camera during three postnatal periods (P3–P6, P7–P10, and P11–P14). Four different maternal behaviors (1: off pups; 2: nursing [arched back and prone]; 3: licking or grooming the pups; 4: self-maintenance) were observed on video, and at every 30 s interval, a snapshot of the behavior that they were engaged in was quantified (60 times in total). The total number of times each behavior occurred was then divided by 60 to determine the percentage of time spent on each behavior.

### 4.9. Tissue Sample Preparation for Histological and Immunohistochemical Analyses

After the behavioral analyses, the mice were deeply anesthetized before being transcardially perfused with ice-cold PBS. Part of each brain sample was immediately frozen using liquid nitrogen and preserved at −80 °C. The remaining brain tissue, nasal mucosa, and lung tissue were collected and fixed overnight at 4 °C with 4% paraformaldehyde diluted in PBS. After the fixation, the tissue was processed for paraffin embedding. Briefly, sections were dehydrated with ethanol, gradually infiltrated with heat-melted paraffin, and embedded in a paraffin block. The nasal mucosa was decalcified before dehydration to remove the nasal and cranial bone. After sectioning at 6 µm thickness, the nasal mucosa and lung sections were routinely stained with hematoxylin and eosin and periodic acid–Schiff (PAS).

### 4.10. Immunofluorescence Analyses

For the immunofluorescence analyses, paraffin-embedded sections were deparaffinized in xylene, rehydrated in 99% ethanol, and subjected to antigen retrieval. For antigen retrieval, the sections were autoclaved in 10 mM citrate buffer for 10 min at 121 °C before being cooled to room temperature. The sections were then permeabilized with Tris-HCl containing 0.1% Triton for 3 min twice and with Tris-HCl without Triton once. Next, the sections were incubated overnight with primary antibodies at 4 °C. The primary antibody details are listed in the Supplementary Table S1. After rinsing, the sections were incubated with Alexa Fluor 488- or 594-conjugated secondary antibodies (1:500; Thermo Fisher Scientific, Rockford, IL, USA) and then mounted with mounting medium including 4′,6-diamidino-2-phenylindole (H1200; Vector, Burlingame, CA, USA). Images were captured using an all-in-one fluorescence microscope (BZ-X800, KEYENCE, Osaka, Japan). For postsynaptic density protein 95 (PSD95) staining, images taken as a Z stack of 13 images, including 0.3 µm pitch, were reconstructed with the full focus function into 1 image using Keyence image analysis software (BZ-X Analyzer software, KEYENCE, Osaka, Japan, https://www.keyence.co.jp/ss/products/microscope/bz-casestudy/example-of-description.jsp, accessed on 6 September 2024).

### 4.11. Golgi–Cox Staining

Modified Golgi–Cox staining was performed using a kit (super Golgi Kit; #003010; Bioenno Lifesciences, Inc., Santa Ana, CA, USA) according to the manufacturer’s instructions. Brain sections were cut using a vibratome at 150 µm thickness. When classifying dendritic spine morphologic types, we used the method described by Li et al. [63].

### 4.12. Microarray

Frozen mouse brain OBs (*n* = 4 per group) were used for the microarray analyses. To obtain a sufficient amount and quality of RNA, the whole OB samples in one group were lysed, pooled, and used as the representative bulk RNA sample for that group. Total RNA was isolated using an RNeasy Mini Kit (Qiagen, Hilden, Germany) before being quantified using an ND-1000 spectrometer (NanoDrop Technologies, Wilmington, DE, USA). The RNA quality was confirmed using a 4150 TapeStation (Agilent Technologies, Santa Clara, CA, USA); the RNA integrity numbers ranged between 8.1 and 8.6. According to the manufacturer’s protocol, total RNA (100 ng) was labeled using a GeneChip WT PLUS Reagent Kit (Thermo Fisher Scientific), hybridized to a Clariom D Array, Mouse (Thermo Fisher Scientific), and scanned using a microarray scanner (Agilent Technologies). The scanned RNA expression data were calculated and standardized using Expression Console software (v1.4, Thermo Fisher Scientific). For standardization, the signal space transformation–robust multi-chip analysis algorithm was used, and its annotation level was set to the gene level. The Database for Annotation, Visualization and Integrated Discovery (DAVID; https://david.ncifcrf.gov, accessed on 6 January 2023) was used to identify enriched gene sets, and heatmap/cluster analysis was conducted using Multiple Experiment Viewer (MeV; https://sourceforge.net/projects/mev-tm4/, accessed on 30 August 2024). The gene expression results were uploaded to the Gene Expression Omnibus repository (Accession number: GSE271274) on the National Center for Biotechnology Information’s homepage (https://www.ncbi.nlm.nih.gov/geo/, accessed on 30 August 2024).

### 4.13. Statistics

All data are presented as the mean ± SEM. The significance of any differences between values was determined using Tukey’s multiple comparison test for the behavioral tests, a two-way analysis of variance with Bonferroni’s multiple comparison test for the maternal nursing behavior study, and the Mann–Whitney *U* test for all other analyses. All data were statistically analyzed using GraphPad Prism 10.2.3 (GraphPad Software, San Diego, CA, USA). Significance was set at *p* < 0.05.

## 5. Conclusions

Independent of maternal allergic changes, postnatal allergic exposure in offspring induces mild allergic rhinitis, eosinophilic infiltration to the OB, and subsequent reactive microgliosis and astrogliosis. As a result, mPFC neural activity projecting from the OB is decreased as a result of impaired OB synaptic formation, leading to impaired mPFC spine formation and maturation and ASD-like behavioral deficits.

## Data Availability

The datasets generated and/or analyzed during the present study will be made available by the corresponding author upon reasonable request from any qualified investigator.

## Supplementary Materials

The supporting information can be downloaded at: https://www.mdpi.com/article/10.3390/ijms251910464/s1.
