# Supplementary material for: Postnatal Allergic Inhalation Induces Glial Inflammation in the Olfactory Bulb and Leads to Autism-Like Traits in Mice
Source: Int J Mol Sci. 2024 Sep 28;25(19):10464. doi: 10.3390/ijms251910464 (PMC11476352; doi:10.3390/ijms251910464)
Supplement: Supplementary file 1 [file ijms-25-10464-s001.zip › ijms-3210247-supplementary.pdf]

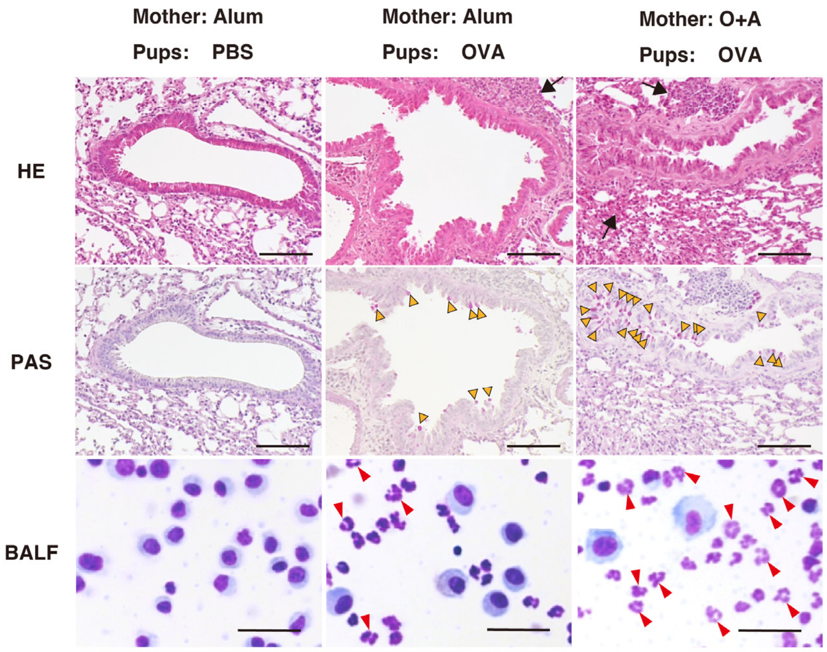

Supplementary Figure S1.

Lung histopathological analyses of dams with allergic exposure. On HE and PAS staining, gradual granulocyte infiltration (black arrows) and mucus production (yellow arrowheads) were observed in the mildly allergic and allergic groups compared with the control group (non-allergic group). BALF sediment analyses indicated a gradual increase in eosinophils (red arrowheads) in the mildly allergic and allergic groups. Scale bars: HE and PAS, 100 µm; BALF, 30 µm.

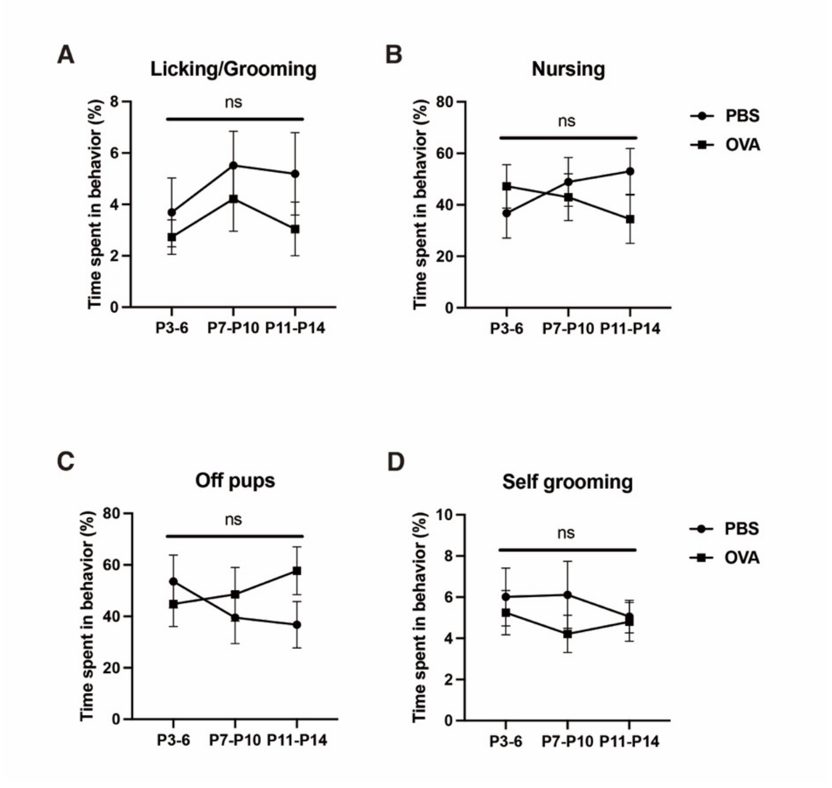

Supplementary Figure S2.

Changes in maternal nursing behavior after allergic exposure in the postpartum dam. During three postnatal periods (P3–P6, P7–P10, and P11–P14), snapshots taken at 30-s intervals during 30 min of video monitoring were classified into one of four behaviors: (A) licking/grooming of their offspring, (B) nursing (arched back and prone), (C) off pups with no nursing, (D) self-grooming behavior (i.e., not for their offspring but for themselves). All values are shown as the mean ± SEM. Two-way analysis of variance with Bonferroni's multiple comparison test was used for the statistical analysis. *n* = 11–15 per group. ns = not significant.

Supplementary Table S1. Antibodies used in the present study

Antibody	Host	Clonality	Dilution	Supplier	Catalog
ECP	rabbit	polyclonal	1:100	Aviscera Bioscience	A00128-03-100
FOSB	rabbit	monoclonal	1:400	Cell signaling technology	#2251
GFAP-Cy3	mouse	monoclonal	1:100	Sigma-Aldrich	C9205
Iba1	rabbit	polyclonal	1:900	Wako	019-19741
PSD95	mouse	monoclonal	1:100	Thermo Fisher Scientific	MA1-046

ECP: eosinophil cationic protein; FOSB: FBJ murine osteosarcoma viral oncogene homolog B; GFAP: glial fibrillary acidic protein; Iba1: ionized calcium-binding adapter molecule 1; PSD95: postsynaptic density protein 95
